# Wound healing in cancer patients under immunotherapy

**DOI:** 10.1097/JS9.0000000000002819

**Published:** 2025-06-27

**Authors:** Pao-Jen Kuo, Pi-Chieh Lin, Ching-Hua Hsieh

**Affiliations:** Department of Plastic Surgery, Kaohsiung Chang Gung Memorial Hospital, Chang Gung University and College of Medicine, Kaohsiung, Taiwan

**Keywords:** immune checkpoint inhibitors, immune-related adverse events, immunotherapy, surgical complications, wound healing

## Abstract

This comprehensive review examines the impact of cancer immunotherapies on wound healing, focusing on recent clinical evidence. While most cancer patients on immunotherapy heal surgical and accidental wounds without significant impairment, immune system alterations can sometimes delay healing or cause unique wound pathologies. Neoadjuvant immunotherapy before complex surgeries has shown higher wound complication rates in some cohorts, suggesting careful patient selection is needed. Immune checkpoint inhibitors don’t typically increase general surgical complication rates, offering reassurance for most procedures. However, immunotherapy can directly cause wounds through immune-related adverse events like bullous pemphigoid or pyoderma gangrenosum, requiring specialized management. For malignant wounds, immunotherapy offers potential benefits by treating the underlying cancer. Management strategies include preoperative planning, careful surgical technique, close postoperative monitoring, and multidisciplinary collaboration. Despite adding complexity to wound healing, immunotherapy’s cancer control benefits typically outweigh risks. With appropriate strategies and vigilance for impaired healing or unusual wound presentations, most patients can undergo surgery safely while continuing immunotherapy. Further research is needed to develop evidence-based guidelines for perioperative immunotherapy management.

## Introduction

Advances in immunotherapy have revolutionized cancer treatment over the past decade, improving survival in many malignancies. Immune checkpoint inhibitors (ICIs) such as anti-programmed cell death 1 (PD-1), anti-programmed death-ligand 1 (PD-L1), and anti-cytotoxic T-lymphocyte antigen 4 (CTLA-4) antibodies are now standard of care for numerous cancers^[1,2]^. Likewise, adoptive cellular therapies, including as chimeric antigen receptor (CAR) T-cell therapy and tumor-infiltrating lymphocytes (TILs), together with cytokine therapies like interleukin-2 (IL-2), have expanded therapeutic options in oncology^[3,4]^. As a result, a growing number of cancer patients on these immunotherapies present with wounds—whether surgical incisions, chronic ulcers, or tumor-related wounds—during their cancer journey. This convergence raises critical questions about how immunotherapy impacts wound healing in clinical practice.

Wound healing is a complex process requiring a finely tuned immune response. Cancer patients often have risk factors for impaired healing, including older age, malnutrition, steroid use, and prior treatments. Traditionally, oncologic therapies like cytotoxic chemotherapy or anti-angiogenic drugs (e.g., bevacizumab) are known to delay wound healing by suppressing cell proliferation or blood vessel growth^[1]^. In contrast, immunotherapies stimulate immune activity rather than directly inhibiting regenerative pathways. The net effect of immunotherapy on wound repair is not yet fully understood. Early clinical observations have been mixed—some reports suggest immunotherapy can be given safely during the perioperative period^[5]^, whereas others have noted higher rates of wound complications in certain settings^[1]^. Given the increasing overlap of surgery, wound care, and immunotherapy in oncology, a thorough review of current evidence is warranted.

This article reviews the impact of cancer immunotherapies on wound healing, emphasizing clinical trial data and case reports from the past 5 years. We examine wound outcomes in patients receiving ICIs, chimeric antigen receptor T-cell (CAR T-cell) therapy, cytokines, and other immunotherapies. Both acute surgical wounds and chronic wounds (including non-healing ulcers and malignant fungating wounds) are discussed. We also explore underlying mechanisms for delayed healing in this population and highlight management strategies. By synthesizing recent clinical evidence, this review aims to guide oncology and wound care specialists in optimizing outcomes for cancer patients on immunotherapy. Following PRISMA 2020 recommendations^[6]^, we conducted a systematic literature search in PubMed/MEDLINE, Embase, Scopus, and Web of Science from database inception to 1 March 2025. The search string combined controlled vocabulary (MeSH/Emtree) and free-text terms for the three core concepts:^[1]^ cancer OR malignancy OR neoplasm,^[2]^ immunotherapy OR “immune checkpoint inhibitor” OR PD-1 OR PD-L1 OR CTLA-4 OR CAR-T OR cytokine therapy, and^[3]^ wound healing OR surgical wound OR chronic ulcer OR skin ulcer OR complication. Boolean operators and truncation were applied (e.g., (cancer OR neoplasm) AND (immunotherapy OR ipilimumab OR pembrolizumab) AND (“wound healing” OR ulcer)), and filters were not used to avoid inadvertent study loss. Inclusion criteria: (i) peer-reviewed original research (randomized trials, cohort or case–control studies, large case series ≥10 patients); (ii) adult or pediatric humans with malignancy receiving systemic immunotherapy; and (iii) quantitative or qualitative data on wound-related outcomes (e.g., infection, dehiscence, chronic ulcer behavior). Exclusion criteria: non-English articles, single-patient case reports, in-vitro/animal studies, conference abstracts without full data, and duplicate publications. Two reviewers independently screened titles/abstracts, then full texts, resolving disagreements by consensus. Risk of bias was assessed with RoB 2 for randomized controlled trials and ROBINS-I for non-randomized studies; results informed narrative synthesis and sensitivity analyses, thereby mitigating selection bias and enhancing the robustness of conclusions. The use of artificial intelligence (AI) assistance in the manuscript drafting is in accordance with the TITAN 2025 guidelines to ensure full transparency of AI involvement^[7]^. AI language-assistance (OpenAI GPT-4o) was employed for spelling, grammar, and reference cross-checking. The complete prompt transcripts for ChatGPT were as follows: Determine whether there is an abbreviation without a full name before it, or whether the full name is used again in the text despite that an abbreviation has previously been provided. Then, act as a reviewer to identify any errors in the referenced reference and its associated description in the text. Check the consistency of in-text citations and metadata (e.g., author, title, journal), as well as whether study descriptions accurately match the cited publications. Report all minor formatting, grammatical, and citation issues.

This review adds to the recent existing literature by providing a comprehensive overview of wound healing in cancer patients receiving immunotherapy, addressing a previously underexplored intersection of oncology and surgical care. It highlights new insights into how immunotherapy influences wound healing dynamics, with evidence suggesting that ICIs can alter normal tissue repair and potentially increase wound complication rates. These findings carry important implications for surgical planning, including careful timing of immunotherapy relative to surgery and heightened vigilance for healing complications. The review also delineates the role of immune-related adverse events (irAEs) such as inflammatory toxicities and steroid requirements in impairing wound repair and recovery.

## Overview of wound healing in cancer patients

Wound healing proceeds in overlapping phases—hemostasis, inflammation, proliferation, and remodeling—orchestrated by immune cells, cytokines, and stromal cells. In cancer patients, this process can be compromised by both patient- and treatment-related factors. Advanced age and comorbidities such as diabetes or vascular disease are common and contribute to impaired healing and higher infection risk. Malignancy itself can impair nutritional status and immune function, delaying tissue repair. Moreover, many cancer patients have a history of chemotherapy or radiation, which can cause tissue fibrosis and reduce healing capacity^[8]^. Prior radiation therapy, for example, is known to predispose to chronic radiation ulcers that heal poorly due to fibrosis and poor vascularity^[8]^.

**HIGHLIGHTS**

- Immunotherapy generally does not impair surgical wound healing, though neoadjuvant use may increase complication risks in complex surgeries.
- Immune-related skin toxicities from immunotherapy, like bullous pemphigoid, can cause severe chronic wounds needing immunosuppressive treatments.
- Successful immunotherapy shrinks malignant wounds but may initially worsen wounds through rapid tumor necrosis, requiring specialized multidisciplinary wound care.

In addition, the interplay between radiation therapy and immunotherapy can influence postoperative wound behavior^[9]^. Pre-clinical work shows that fractionated radiation impairs fibroblast proliferation and microvascular integrity, prolonging the inflammatory phase and delaying collagen maturation^[8,10]^. Clinically, patients receiving neoadjuvant chemoradiation followed by immunotherapy demonstrate higher risks of wound dehiscence and surgical-site infection (SSI)—up to a twofold increase in head-and-neck free-flap cohorts^[1]^. Nevertheless, modern conformal or hypofractionated protocols (≤30 Gy) combined with PD-1 blockade have achieved acceptable wound complication rates (<10%) in soft-tissue sarcoma and rectal cancer series when surgery is delayed ≥4 weeks after the last radiation fraction^[11–13]^. Consequently, scheduling surgery 4–6 weeks after radiation, carefully managing irradiated tissue, and implementing multidisciplinary monitoring should be prioritized to mitigate late necrosis or fistula development.

Notably, prolonged or high-dose corticosteroid therapy—sometimes required in oncology for symptom management or as part of cancer regimens—is a well-known risk factor for delayed wound healing. Steroids impede inflammatory cell recruitment and collagen deposition, which can result in frail wounds and dehiscence. In the context of immunotherapy, this is salient because irAEs often necessitate corticosteroid treatment. Thus, cancer patients under immunotherapy may experience impaired wound healing not only from the drugs themselves but also from the immunosuppressive medications used to manage their side effects^[14,15]^.

Cancer patients may be present with various wound types. Surgical wounds are common in oncology, from tumor resections or biopsy procedures. Chronic wounds such as pressure ulcers or non-healing surgical sites can develop in patients with advanced disease or frailty. Additionally, some tumors cause malignant fungating wounds—ulcerative, malodorous lesions where the tumor infiltrates the skin. Managing these cancer-related wounds is challenging; they often have continuous tumor growth, infection, and poor healing capacity. With the advent of immunotherapy, clinicians are encountering new scenarios, such as patients on ICIs undergoing major surgeries or experiencing unusual wound complications related to immune activation. Understanding baseline wound healing challenges in oncology provides a foundation to appreciate the added complexity introduced by immunotherapies.

## Cancer immunotherapy: mechanisms and modalities

Cancer immunotherapies harness or modulate the immune system to attack tumors. The most widely used class is ICIs, which release brakes on T-cells. PD-1/PD-L1 inhibitors (e.g., pembrolizumab, nivolumab, atezolizumab) and CTLA-4 inhibitors (e.g., ipilimumab) have demonstrated durable tumor responses by enhancing cytotoxic T-cell activity in the tumor microenvironment^[1]^. These agents, however, also carry a risk of irAEs due to loss of self-tolerance. The skin and gastrointestinal tract are among the most frequently involved organ systems in ICI toxicity^[16]^. Common cutaneous irAEs include rash, pruritus, and vitiligo, but more severe inflammatory dermatoses can occur^[17–19]^. Combination immunotherapy (e.g., anti-PD-1 + anti-CTLA-4) tends to produce earlier and more severe toxicities than single-agent therapy^[20]^, which is relevant when considering wound healing and tissue inflammation.

Beyond ICIs, adoptive cellular therapies represent another pillar of immunotherapy. Chimeric antigen receptor T-cell therapy involves engineering a patient’s T lymphocytes to target cancer antigens. Chimeric antigen receptor T-cells are approved for refractory leukemias, lymphomas, and myeloma and yield high remission rates. However, CAR T recipients undergo lymphodepleting chemotherapy and often develop cytopenia, B-cell aplasia, and hypogammaglobulinemia that can persist for months^[21]^. They are consequently at risk for infections, which could adversely affect wound healing or surgical outcomes^[22,23]^. Other cellular therapies include TILs expanded ex vivo and reinfused to attack solid tumors, an approach showing promise in melanoma^[24]^. TIL therapy requires surgical harvesting of tumor tissue, introducing a wound that must heal while the patient later receives high-dose IL-2 and lymphodepleting chemo as part of the protocol.

Cytokine therapies were among the earliest immunotherapies in oncology. High-dose IL-2 can induce long-term remissions in melanoma and renal cell carcinoma by broadly activating immune effectors, but its usage has declined due to severe toxicity like capillary leak syndrome or organ dysfunction^[25]^. Interferon-α, another cytokine, was used as adjuvant therapy in melanoma; it boosts immune surveillance but causes chronic flu-like symptoms and fatigue^[26]^. While these cytokines activate immunity, they can also cause systemic side effects (e.g., hypotension with IL-2) that may indirectly impair tissue perfusion or healing. Finally, oncolytic virus therapy (e.g., talimogene laherparepvec, a modified herpesvirus) is a localized immunotherapy injected into tumors. It causes tumor cell lysis and provokes an immune response^[27,28]^. Oncolytic virus treatment of cutaneous tumors often leads to local inflammation and ulceration at injection sites as tumors necrose, essentially creating an acute wound that must heal over time^[27,28]^. Table 1 provides an overview of how key immunotherapy modalities might influence wound healing, through both direct immunologic mechanisms and indirect effects such as necessary adjunct medications. Table 2 summarizes key clinical studies examining surgical outcomes in cancer patients on immunotherapy. Understanding these potential mechanisms is crucial for interpreting clinical outcomes in patients on immunotherapy who develop wounds.

**Table 1:** Provides an overview of how key immunotherapy modalities might influence wound healing, through both direct immunologic mechanisms and indirect effects such as necessary adjunct medications. Understanding these potential mechanisms is crucial for interpreting clinical outcomes in patients on immunotherapy who develop wounds

Mechanism or Factor	Potential Impact on Wound Healing	Evidence/Notes
Heightened T-cell activity (via PD-1/CTLA-4 blockade)	Prolonged inflammation in wound, tissue damage to normal cells	PD-1/PD-L1 pathway normally helps resolve inflammation; blocking it may delay healing^[23]^.
Macrophage polarization shift (less M2 pro-healing)	Impaired granulation and remodeling phases	PD-L1 expression on fibroblasts promotes M2 macrophages; PD-1/PD-L1 inhibitors may hinder this transition^[23]^.
Autoimmune skin reactions (e.g., bullous pemphigoid, vasculitis)	Direct damage to skin integrity, chronic ulcerations	ICI-induced bullous pemphigoid causes subepidermal blisters and requires immunosuppressive therapy^[25]^.
Tumor necrosis and ulceration (rapid tumor response)	Formation of wound or fistula from tumor tissue breakdown	Rapid tumor regression on immunotherapy can leave open defects^[29]^, e.g., fistula formation in gastrointestinal tumors.
Infection risk from immune dysregulation	Secondary wound infections, impaired healing	CAR T-cell therapy leads to B-cell aplasia and hypogammaglobulinemia, increasing infection susceptibility^[14]^.
Corticosteroid use for irAEs	Slowed collagen deposition, wound dehiscence, infection risk	High-dose steroids given to manage colitis or dermatitis blunt wound healing responses^[14]^.
Anti-angiogenic effects (indirect or combined therapy)	Reduced neovascularization in wound bed	Some patients receive immunotherapy with anti-VEGF agents; anti-VEGF is known to impair wound healing^[1]^.

CAR T-cell = Chimeric Antigen Receptor T-cell; CTLA-4 = Cytotoxic T-Lymphocyte Antigen 4; ICI = Immune Checkpoint Inhibitor; irAE = Immune-related Adverse Event; PD-1 = Programmed Cell Death-1; PD-L1 = Programmed Death-Ligand 1; VEGF = Vascular Endothelial Growth Factor.

**Table 2:** Summarizes key clinical studies and reports from recent years examining surgical outcomes in cancer patients on immunotherapy. It highlights the heterogeneity of findings and underscores that while most data are reassuring, vigilance is needed, especially for patients receiving neoadjuvant immunotherapy in anatomically complex surgeries

Study (Year)	Patient Population and Immunotherapy	Key Findings on Wound/Surgical Outcomes
Mays *et al* ^[2021,^ ^1]^	132 head/neck cancer patients with free flap reconstruction; many on neoadjuvant or adjuvant anti-PD-1	Neoadjuvant immunotherapy associated with higher major wound complication rate (OR ~ 3.7); predominantly recipient site dehiscence/necrosis. Adjuvant-only ICI did not show increased risk.
Tang *et al* ^[2024,^ ^30]^	7674 high-risk surgery patients with various cancers in intensive care units, 247 had ICI ≤6 months pre-operation (mostly PD-1/PD-L1)	No significant difference in composite serious complication rate between ICI-exposed and matched controls (35% vs 32%). No increase in 30-day mortality or length of stay. Slight increase in vasopressor requirement post-operation in ICI group.
Philips *et al* ^[2024,^ ^31]^	Retrospective comparison of neoadjuvant ICI vs no ICI in patients with mixed cancer surgeries	Neoadjuvant checkpoint inhibitors did not increase overall surgical complication rates after adjusting for confounders. Supports safety of surgery with prior ICI exposure.
Huang *et al* ^[2024,^ ^2]^	6 RCTs (2941 patients) resectable NSCLC; neoadjuvant or perioperative PD-1/PD-L1 ± chemotherapy vs chemotherapy	Perioperative immunotherapy improved pathologic response and survival. Surgical resection rates were similar or better with ICI. Post-op complication rates such as pneumonia and bronchial fistula rare were comparable between arms; one trial noted a higher fistula rate (25%) with ICI, but others were 0–3%.
Shapiro *et al* ^[2023,^ ^32]^	75 patients metastatic renal cancer underwent cytoreductive nephrectomy after ICI combo therapy	90-day major complication rate only 3%; no deaths. Wound healing was not problematic despite concerns of desmoplastic reaction. Concluded post-ICI surgery is feasible with low complications in experienced centers.
Yip *et al* ^[2023,^ ^33]^	Multicenter study of nephrectomy after ICI	Similarly found nephrectomy post-ICI is feasible. Complication profile akin to that of primary surgery; immunotherapy did not significantly worsen wound outcomes.
O’Neill *et al* ^[2022,^ ^34]^	48 patients on ICIs undergoing 60 dermatologic excisional Mohs surgeries	Low complication rates: 4% minor infection, no significant wound dehiscence. Healing proceeded normally in all cases. Concludes that minor surgeries are safe during ICI therapy.
Case reports (2018–2023)	Various (colorectal, melanoma, etc.)—e.g., fistula formation after PD-1 therapy	Highlight unique complications: immunotherapy-induced tumor necrosis causing wound/fistula requiring surgery. Emphasizes need for monitoring and possible surgical standby when treating large tumors with ICIs.

NSCLC = Non-Small Cell Lung Cancer; OR = Odds Ratio; RCTs = Randomized Controlled Trials.

## Immunotherapy effects on wound healing: from biology to clinical observations

### Immune activation and wound healing dynamics

Effective wound healing requires a balanced immune response—enough inflammation to prevent infection and clear debris, but timely resolution to allow tissue regeneration. Immunotherapies, by design, tilt this balance toward heightened immune activity. Preclinical studies have begun to illuminate how this altered immune milieu affects healing. Notably, the PD-1/PD-L1 checkpoint, famous for its role in tumor immune evasion, also appears to function in wound sites as a modulator of inflammation resolution^[35]^. PD-L1 expression in wounds: Recent research demonstrated that fibroblast-like cells in granulation tissue upregulate PD-L1, which helps create an “immunosuppressive” microenvironment in wounds that promotes the switch from pro-inflammatory (M1) to pro-healing (M2) macrophages^[29]^. Blocking PD-L1 or PD-1 could therefore disturb this switch. Wang *et al* (2022) found that PD-L1 on wound fibroblasts is a positive regulator of healing, and genetic or pharmacologic interruption of PD-1/PD-L1 signaling led to delayed wound closure in their models^[29]^. These findings suggest that ICIs might inherently predispose wounds to remain longer in an inflammatory state. However, although PD-L1 appears to foster normal wound repair via inflammation resolution, as PD-L1 knockout mice have delayed wound closure^[36]^, it is not yet known how PD-1/PD-L1 blockade affects healing in patients. Furthermore, no reliable biomarkers exist to predict which patients on ICIs may develop wound healing complications, and the differential impact of immunotherapy on healing across different tissues remains unclear, creating a research gap that requires further investigation.

Exuberant T-cell activity can also cause off-target tissue damage. ICIs unleash T-cells that may attack not only tumor cells but also normal tissues expressing cross-reactive antigens. In skin, this can manifest as interface dermatitis, lichenoid reactions, or even vasculitic changes that compromise microvascular supply to healing tissue. A dramatic example is bullous pemphigoid induced by ICIs. BP is an autoimmune blistering disorder typically seen in elderly patients, wherein antibodies target basement membrane proteins, causing subepidermal bullae and erosion. ICI-induced BP has been increasingly reported, usually after a few months of PD-1 or PD-L1 inhibitor therapy^[37]^. While rare (~1% or less of patients), it poses a significant wound care challenge: patients develop widespread, chronic erosions that are essentially non-healing wounds requiring immunosuppressive treatment^[31]^. One single-institution study noted that ICI-induced BP led to substantial morbidity and often necessitated therapy discontinuation and systemic treatments (steroids, tetracyclines, or dupilumab) to control the skin lesions^[38]^. This illustrates how augmenting the immune system can paradoxically result in severe wound-like toxicities.

Another immune-mediated wound pathology is pyoderma gangrenosum (PG), a neutrophilic dermatosis characterized by painful ulcerative lesions. Pyoderma gangrenosum can be triggered by immune dysregulation and has been associated with medications, including immunotherapies. There are case reports of PG developing after initiation of checkpoint inhibitors, possibly related to an exaggerated neutrophil-driven inflammatory response in the skin^[39]^. For instance, a patient receiving anti-PD-1 therapy for melanoma developed a rapidly enlarging ulcer with undermined borders at a surgical site, consistent with post-surgical PG^[32]^. Checkpoint inhibitor therapy was identified as a potential precipitant, given its broad activation of T-cells and cytokines that may have unmasked an autoinflammatory condition^[39]^. Management requires immunosuppressive therapy and wound care, underscoring that these irAEs can significantly complicate the clinical course.

On the other hand, immunotherapy’s effect is not universally detrimental to wounds—in some scenarios it may aid healing indirectly by controlling infection or tumor burden. Heightened immune surveillance could, in theory, reduce SSIs. Unlike cytotoxic chemotherapy, ICIs do not cause neutropenia or directly inhibit cell proliferation in normal tissue^[33]^, so the fundamental capacity for cell division and collagen synthesis remains intact. Additionally, successful immunotherapy can shrink tumors that are causing or infiltrating wounds, thereby removing an impediment to healing. An illustrative case involved an elderly patient with a large, ulcerating inguinal metastasis (a fungating tumor mass) that was causing severe pain and immobility^[40]^. The patient’s tumor had high PD-L1 expression, so pembrolizumab immunotherapy was initiated for palliative intent. Within 3 weeks, the tumor rapidly regressed, leaving an open cavity where the mass had been—essentially converting a cancerous wound into a post-tumor wound bed^[40]^. This response obviated the need for palliative radiation, which would likely have further impaired healing, and the wound could then be managed with standard wound care. Such cases demonstrate that by eliminating tumor tissue, immunotherapy can sometimes facilitate the eventual healing of malignant wounds. However, they also highlight a need for careful wound management during tumor regression, as rapid necrosis can produce large defects or fistulas. In one report of a patient with an microsatellite instability-high colorectal tumor, anti-PD-1 therapy led to massive tumor necrosis that resulted in a gastrocutaneous fistula, necessitating surgical intervention^[34]^. Thus, while immunotherapy can promote tumor clearance, the byproducts of that success may be acute wound complications that require multidisciplinary management.

### Immune mediators in wound repair

Wound healing is orchestrated by a dynamic interplay of immune cells and their mediators that drive inflammation, tissue formation, and remodeling^[41]^. In the immediate post-injury phase, innate immune cells (neutrophils, macrophages, and mast cells) are recruited to clear debris and prevent infection, releasing cytokines and growth factors that set the stage for repair^[42,43]^. Mast cells, traditionally known for allergic responses, have emerged as pivotal regulators in this process. Upon injury, skin mast cells degranulate and secrete mediators such as histamine, tumor necrosis factor (TNF)-α, interleukins (IL-6, IL-8), and vascular endothelial growth factor (VEGF), which increase vascular permeability and vasodilation, facilitating the influx of inflammatory cells (e.g., monocytes and neutrophils) to the wound bed^[44]^. Mast cell-derived signals also activate fibroblasts and keratinocytes. For example, fibroblast proliferation and new extracellular matrix deposition are stimulated by mast cell interleukin-4, basic fibroblast growth factor, and VEGF^[44]^, while angiogenesis and re-epithelialization are promoted by other mast cell products, including fibroblast growth factor 2 (FGF-2), platelet-derived growth factor (PDGF), transforming growth factor beta (TGF-β), and nerve growth factor^[44]^. Through these actions, immune mediators ensure effective transition from the inflammatory phase to the proliferative phase of healing. It is important to note that a well-regulated immune response is essential for successful wound repair. This response must be sufficient to combat pathogens and promote repair, while also being controlled enough to prevent collateral injury. While other mast cell products (FGF-2, PDGF, TGF-β, nerve growth factor) promote angiogenesis and re-epithelialization^[45]^. Excess or prolonged inflammation is a major culprit in delayed or pathological healing: persistent high levels of inflammatory cytokines can disrupt normal progression of wound closure, and clinical studies show that limiting such inflammation can reduce fibrosis and scarring^[42]^. Failure to terminate the inflammatory phase due to continued infection or ischemia results in chronic, non-healing wounds characterized by ongoing immune cell infiltration and tissue breakdown^[36,46]^. Mast cells are often implicated in these secondary complications; chronic wounds and fibrotic scars have been associated with increased mast cell accumulation, suggesting that unrestrained mast cell activation may perpetuate inflammatory signaling and aberrant remodeling^[47]^. In diabetic foot ulcers—a paradigm of impaired healing—mast cells appear to stimulate “delayed” inflammation through crosstalk with other cells and the extracellular matrix, which can alter macrophage behavior and diminish healing efficacy^[48]^. Immune checkpoint inhibitors, by unleashing T-cell activity, can create a pro-inflammatory milieu; indeed, preliminary clinical data in head and neck cancer patients indicate that preoperative immunotherapy exposure may correlate with higher rates of wound complications^[1]^. Moreover, targeted therapies used in oncology (for example, anti-VEGF antibodies) are well known to impair angiogenesis and wound healing, highlighting the need for vigilance when managing wounds in immunotherapy-treated patients^[1]^. Overall, the balance of immune mediators is central to proper wound healing. Therapeutic strategies aimed at modulating this balance are being explored—from mast cell stabilizers (e.g., cromolyn) or chymase inhibitors to dampen excessive fibrosis^[47]^, to novel cell and gene therapies designed to provide controlled release of anti-inflammatory factors, and to prevent secondary inflammatory complications^[48]^. Ensuring optimal immune regulation may be especially critical in cancer patients on immunotherapy to promote timely wound healing and minimize delayed complications.

### Clinical outcomes in surgical wound healing under immunotherapy

A key question for oncology surgeons is whether ongoing or recent immunotherapy elevates the risk of postoperative complications, such as wound dehiscence, SSI, or delayed healing. Overall, emerging evidence from retrospective cohorts and clinical trials suggests that performing surgery in patients receiving ICIs is feasible, with acceptable complication rates, but certain subsets and contexts require caution.

A multicenter study of 132 head and neck cancer patients undergoing major resection with free flap reconstruction found that preoperative ICI therapy was associated with significantly higher rates of major wound complications^[1]^. Patients with neoadjuvant ICI exposure were 3.7 times more likely to require invasive intervention for recipient-site wound complications compared to those without exposure. Donor-site complications also trended higher (odds ratio ~ 7, not statistically significant)^[1]^. Postoperative ICI therapy did not increase risk, suggesting timing of exposure is important. While all patients eventually achieved flap healing, those with complications needed additional interventions. The authors concluded that preoperative immunotherapy may increase wound complications and recommended further controlled studies^[1]^.

A recent meta-analysis of six randomized trials (2941 patients) examining perioperative ICIs in resectable non-small cell lung cancer found that immunotherapy improved pathological response rates and survival outcomes without preventing complete surgical resection^[2]^. While immunotherapy groups experienced more grade 3–5 adverse events during treatment, perioperative complications were comparable between arms^[2]^. Postoperative pneumonia ranged from 0 to 5% across studies, and bronchopleural fistula rates were typically 0–3%, with only one outlier trial reporting up to 25%^[49]^. The data indicates that operating after neoadjuvant ICI therapy is feasible and safe in lung cancer patients, with complication rates consistent with historical norms^[30]^. These findings suggest that immunotherapy should not be considered an absolute contraindication to timely surgery, though careful monitoring remains important.

Experience in other tumor types supports these findings. In metastatic renal cell carcinoma, combination immunotherapy (anti-PD-1 plus anti-CTLA-4 or anti-VEGF tyrosine kinase inhibitor) is increasingly first line, sometimes followed by cytoreductive nephrectomy. Initial concerns of dense inflammation or fibrosis complicating surgery have largely been disproven^[50]^. A multicenter study of 75 post-immunotherapy nephrectomies reported low intraoperative (4%) and predominantly minor postoperative complication rates (25%), with only 3% major complications (Clavien ≥ III), no increased wound healing issues, and zero 90-day mortality^[51]^. Other centers confirm no significant rise in surgical difficulty or wound complications after immunotherapy^[52,53]^. Overall, surgeries expected to be complicated by immunotherapy-related fibrosis have yielded favorable outcomes.

Small-scale studies in other surgical domains further support that ICIs do not dramatically worsen wound outcomes. In dermatologic surgery, for instance, a retrospective study examined 48 patients on ICIs who underwent 60 dermatologic as excisional Mohs surgeries for skin cancers^[54]^. Postoperative complications were infrequent: only ~ 4% of cases had an infection and ~ 2% had significant wound pain requiring an emergency visit^[54]^. There were no cases of wound dehiscence or failure to heal reported. Although these procedures are minor compared to visceral surgery, the data suggest that even under active systemic immunotherapy, small acute wounds can heal without incident. Additionally, a report on primary head and neck cancer patients receiving adjuvant immunotherapy (pembrolizumab) after lymph node dissection noted that wound complications like seromas or delayed healing were comparable to historical rates prior to the immunotherapy era^[55]^.

An area of uncertainty is the optimal timing of surgery relative to immunotherapy dosing. In clinical trials, protocols often impose a window (e.g., surgery 3–6 weeks after last neoadjuvant ICI dose) to allow acute drug-related inflammation to subside. Outside trials, practices vary—some surgeons pause ICIs around the time of major surgery, while others proceed if the patient is due for therapy. Elias *et al* found that among various cancer surgeries performed on ICI therapy, there was no clear increase in complications and concluded that ICIs “likely do not need to be stopped in the perioperative setting”^[5]^. Current practice is evolving as safety data accumulate. In general, for elective surgeries, it is common to schedule the operation at a nadir of immunotherapy activity (e.g., just before the next cycle is due) and to resume therapy once the wound has shown adequate initial healing (typically 2–4 weeks post-operation), assuming no contraindicating complications. No formal guidelines exist yet, so decisions are individualized. What is evident is that with careful patient selection and perioperative management, even major surgery can be performed on patients actively receiving immunotherapy, with outcomes comparable to those not on these agents^[30]^.

### Chronic and atypical wounds in immunotherapy patients

Beyond acute surgical wounds, clinicians must also manage chronic wounds in cancer patients who are receiving immunotherapy. Chronic wounds—defined as those that do not heal in an orderly time frame (e.g., beyond 3 months)—include pressure ulcers, venous stasis ulcers, diabetic foot ulcers, and long-standing surgical wounds. In patients with cancer, chronic wounds may arise from prolonged immobility, poor nutrition, or treatment complications. The literature specifically addressing chronic wound healing under immunotherapy is sparse; however, we can extrapolate potential considerations from what is known about immunotherapy’s immunologic effects and reported adverse events.

While immune activation might theoretically help chronic wounds by controlling infection, evidence suggests the opposite effect. Chronic wounds are already stuck in inflammation, and checkpoint inhibitors could worsen this state^[29]^. Though no clinical studies confirm detrimental effects in cancer patients on immunotherapy, emerging research indicates that enhancing (not blocking) PD-1/PD-L1 signaling might actually promote wound healing by reducing inflammation^[29,56]^. This suggests that PD-1/PD-L1 inhibitors could potentially impair chronic wound healing. For patients on these therapies with existing chronic ulcers, focused wound care interventions like debridement may be necessary, as immunotherapy is unlikely to help and might slightly hinder healing.

Immunotherapy shows promise for malignant wounds (those caused by tumor infiltration), common in advanced breast cancer, head/neck cancers, and melanoma. While traditional treatment focuses on palliative measures rather than healing, immunotherapy can treat the underlying cancer, potentially improving these wounds as tumors shrink. Case reports document ulcerating lesions from melanoma^[40]^, cutaneous squamous cell carcinoma^[57]^, and Merkel cell carcinoma^[58]^ substantially healing after anti-PD-1 therapy eliminated the tumor burden. In one series, ulcerated Merkel cell tumors often completely re-epithelialized after pembrolizumab treatment^[58]^. For malignant wounds, immunotherapy serves both for cancer control and wound improvement. However, careful wound care remains essential during tumor regression to manage exudate and prevent sepsis. Large cavities resulting from resolved metastases may require surgical closure or reconstruction once cancer is in remission.

Immunotherapy can create chronic wound scenarios through immune-related dermatologic adverse events. These include bullous pemphigoid, severe psoriasis or lichenoid dermatitis flares (sometimes progressing to erythroderma or fissured plaques requiring wound care), and rare cutaneous vasculitis causing leg ulcerations^[59–61]^. Management typically requires withheld or permanently discontinued immunotherapy^[60]^ and administering immunosuppressants like high-dose steroids or infliximab—paradoxically reversing the immunotherapy’s effect to treat its complications^[17,61]^. Multidisciplinary care between dermatologists, wound specialists, and oncologists is crucial to balance cancer treatment with wound management. Some evidence suggests that patients can safely resume immunotherapy after successful treatment of skin adverse events (e.g., using dupilumab for ICI-induced bullous pemphigoid)^[38]^. Decisions must be individualized based on cancer control needs and wound complication severity.

Lastly, it is worth noting the intersection of immunotherapy and infection in wounds. While ICIs do not classically immunosuppress patients, some patients become functionally immunocompromised due to high-dose steroids or other immunosuppressants used for irAEs. Additionally, therapies like CAR T-cells create periods of neutropenia and B-cell aplasia^[21]^. Therefore, a cancer patient on immunotherapy can still be at risk for wound infection or poor healing if their immune system is temporally weakened by these effects. Clinicians should maintain vigilance for infection in wounds and treat promptly with antibiotics and appropriate surgical debridement when needed, as they would in any immunosuppressed patient.

Notably, chronic wound pathophysiology is characterized by unresolved inflammation that can persist beyond the initial healing, meaning that even a “closed” ulcer may still harbor active inflammatory and proteolytic processes^[62]^. Such prolonged, dysregulated inflammation disrupts normal remodeling and may precipitate late wound breakdown or aberrant scar formation months later^[63]^. In addition, chronic ulcers often occur in a milieu of microvascular compromise; impaired angiogenesis and perfusion (for example, due to diabetes or prior radiation) leave the repaired tissue relatively ischemic^[64]^. This ischemia, when combined with an overactive fibrotic response, can produce scar tissue that is less flexible and poorly vascularized, making the area vulnerable to ulcer recurrence or dehiscence under stress^[65]^. Notably, in patients receiving immunotherapy, a heightened immune milieu may exacerbate these issues—ICIs have been associated with increased wound healing complications^[1,66,67]^ and even immune-related vasculitic ulceration presenting months after initial closure^[68]^. In light of these factors, long-term vigilance and adjunct measures to temper chronic inflammation and optimize perfusion are essential to prevent delayed wound complications in this high-risk population.

Table 3: Comparison of wound types and the impact of immunotherapy in cancer patients. Different wound scenarios pose unique challenges in the context of immunotherapy.

**Table 3:** Contrasts how different wound types might be affected by immunotherapy in cancer patients, highlighting the distinct considerations for each

Wound Type	Characteristics in Cancer Patients	Impact of Immunotherapy and Considerations
Acute surgical wound	Clean or clean-contaminated incision from cancer surgery (e.g., resection, flap reconstruction). Healing typically by primary intention.	Checkpoint inhibitors: Generally heal well, though neoadjuvant ICI may increase dehiscence risk in high-tension or poorly vascularized wounds^[1]^. Care: standard SSI prevention; consider timing immunotherapy doses around surgery. CAR T or chemo/immunotherapy combos: If neutropenia or thrombocytopenia present, higher infection/bleeding risk—ensure counts optimized pre-operation.
Chronic non-healing wound	Pressure ulcers, diabetic foot ulcers, or non-healing surgical sites. Often in older, debilitated patients; prolonged inflammatory phase.	Immunotherapy likely offers no direct benefit to healing; excessive inflammation may even perpetuate non-healing state^[23]^. Monitor for flares of inflammation. Manage with aggressive wound care such as debridement. Optimize nutrition. If patient develops an irAE requiring steroids, anticipate further delays in healing and higher infection risk^[50]^
Malignant (fungating) wound	Ulcerative tumor lesions with cancerous tissue, foul odor, drainage (e.g., breast, head/neck tumors breaking through skin). Healing only if tumor is eradicated.	Potential positive impact: Successful immunotherapy can shrink the tumor and allow wound to granulate and epithelialize^[29]^. Marked tumor regression may convert lesion into a healing wound. Challenges: During response, rapid tumor necrosis can enlarge the wound or cause fistulas^[36]^. Requires diligent wound care, infection control, and sometimes surgical debridement. If partial response, remaining tumor can still impede full healing.
Immune-related wound (irAE)	Wounds resulting from immunotherapy toxicity: e.g., autoimmune bullae/erosions (bullous pemphigoid), ulcerative neutrophilic dermatoses (pyoderma gangrenosum), or severe mucocutaneous reactions. These are often chronic until underlying process controlled.	Caused directly by immune hyperactivity. Immunotherapy continuation usually paused due to severity. Management: immunosuppressive therapies (high-dose corticosteroids, IVIG, cyclosporine or biologics) to halt the destructive immune process^[25]^; supportive wound care such as topical therapies and dressings. Healing can be slow; risk of secondary infection is high. Multidisciplinary input is needed. In some cases, immunotherapy can be cautiously resumed with prophylactic measures if cancer control is critical and the wound is improving^[26]^.

irAE = Immune-related Adverse Event; IVIG = Intravenous Immunoglobulin

## Management strategies and clinical implications

Perioperative management recommendations include careful timing of surgery relative to immunotherapy—while early protocols held ICIs for ~3–5 weeks preoperatively, recent evidence indicates that continuing ICIs perioperatively does not impair wound healing^[69]^. If high-dose corticosteroids are required for irAEs, a gradual taper is advised^[17,70]^, and close postoperative wound monitoring is recommended to detect any delayed healing or complications related to immunotherapy^[1]^. Given the above insights, managing wound healing in cancer patients on immunotherapy calls for proactive, multidisciplinary strategies:

**Preoperative planning**: When a patient on immunotherapy is scheduled for surgery, a collaborative approach between the surgical and oncology teams is important. Factors to consider include the timing of the last immunotherapy dose, the patient’s immune-related side effect status, and overall immune system condition. Many clinicians aim to schedule elective surgeries at a point when the patient is not acutely experiencing an irAE or is not immediately post-infusion of immunotherapy (to minimize any peak inflammation). For instance, in a patient receiving pembrolizumab every 3 weeks, a surgery might be done 2–3 weeks after the last dose, giving some buffer time. There is currently no evidence-based mandatory washout period for ICIs, but these pragmatic approaches are used. Importantly, immunotherapy should not be indefinitely withheld without cause, as losing cancer control could be detrimental. If wound healing concerns are present (e.g., a marginally healing biopsy site while on therapy), short delays or breaks can be considered until the wound is stable. The decision must balance oncologic urgency with surgical healing.

Risk factors like diabetes or malnutrition should be optimized pre-surgery, as in any patient. If a patient is on corticosteroids for an immune toxicity at the time surgery is needed, some surgeons will delay elective procedures until steroid dose is tapered to a safer level (e.g., ≤ 10 mg prednisone daily) to reduce infection and dehiscence risk. If surgery is emergent and high-dose steroids are on board, strategies include stress-dose steroids to avoid adrenal crisis but also maximizing local wound measures (extra sutures, drains, abstaining from tension where possible) and antibiotic prophylaxis, given the impaired healing environment.

**Intraoperative techniques**: Surgeons operating on patients who have received immunotherapy may take additional precautions in wound closure. Meticulous technique to minimize tension and ensure good perfusion of the skin flaps is critical—although that is standard for any surgery, it becomes even more important if one worries about an exuberant inflammatory reaction impairing healing. Some head and neck surgeons advocate for the use of vascularized tissue flaps to reconstruct defects in patients who had neoadjuvant ICIs, reasoning that well-vascularized tissue will heal better even in the face of inflammation^[1]^. Indeed, in the Mays *et al* study, despite higher complication rates, ultimately all free flaps were salvaged, suggesting that robust blood supply can overcome some deleterious effects^[1]^. The use of adjuncts like hyperbaric oxygen therapy in difficult wounds could also be considered, though no specific data exists for immunotherapy patients.

If the patient’s surgery is being done after a major tumor response to immunotherapy, surgeons should be prepared for anatomical changes. Dense immune cell infiltration might make tissues appear fibrotic or planes fused which are similar to post-radiation surgery, though studies did not find this significantly hindered resection^[51,60,61]^. Nonetheless, gentle handling of tissues and thorough clearance of any necrotic tumor debris will help create a cleaner wound bed that can heal.

**Postoperative care and monitoring**: Monitor wounds closely after surgery. Address dehiscence, infection, or necrosis immediately through drainage, antibiotics, or plastic surgery consultation. Immunotherapy patients may still show normal infection signs, as ICIs don’t suppress acute phase reactants. Some centers use prophylactic antibiotics more liberally for immunotherapy patients with prosthetic implants or contaminated surgeries and restart immunotherapy individually. If wounds heal well by 2-week follow-up, immunotherapy can usually resume to prevent cancer recurrence. Evidence shows that immunotherapy often continues safely perioperatively^[5]^. For significant complications, delay immunotherapy until wound stabilizes. If extended delay needed, consider alternative cancer treatments to maintain control.

**Managing chronic or complex wounds**: Standard wound care remains essential for cancer patients on immunotherapy with chronic wounds: debride devitalized tissue, maintain moisture balance with advanced dressings, offload pressure, and treat infections. No evidence supports modifying protocols specifically for immunotherapy patients. Monitor for unusual features like violaceous undermined borders or excessive granulation, which may indicate immunologic processes such as PG^[39]^. Biopsy atypical wounds to distinguish between recurrent cancer, immune phenomena, and benign ulcers. For malignant wounds, oncology and palliative wound care teams should collaborate. As tumors respond to immunotherapy, wound characteristics can change rapidly, requiring adaptation from odor-control measures to granulation support. Significant size reduction may create surgical closure opportunities previously unavailable. Regular reassessment and patient education ensure prompt reporting of changes.

**Immunotherapy toxicity management**: If an irAE is causing wound formation (like bullous eruptions or severe rash), aggressive management of the irAE is the priority. Current guidelines for dermatologic irAEs recommend early dermatology consultation and initiating therapies such as systemic steroids for extensive blistering or ulcerative reactions^[17]^. Interruption of the immunotherapy drug is usually mandated in severe grades 3–4 skin toxicities until the skin heals to at least grade 1^[71,72]^. In refractory cases of ICI-induced BP, the use of steroid-sparing agents such as dupilumab, which targets the T-helper 2 cytokines that drive BP, has demonstrated efficacy, enabling some patients to resume their anticancer therapy safely^[38,60,73]^. During these periods, wound care teams should manage the resultant wounds similar to burns or chronic ulcers—using atraumatic dressings, infection prevention, and pain control. The psychological impact on patients should not be overlooked: developing a painful chronic wound from a cancer therapy can be demoralizing. Providing reassurance that these side effects are treatable and, when possible, that immunotherapy can be restarted, if that is an option, helps maintain adherence to cancer care in the long run.

**Cell-based regenerative strategies**: Minimally manipulated autologous cell products—such as point-of-care bone-marrow mononuclear concentrates, adipose-derived stem cells (ADSCs), and keratinocyte-fibroblast suspensions—are under active investigation to shorten healing time in oncology patients whose wounds are slowed by surgery, radiation, or checkpoint blockade^[74–78]^. These cell mixtures deliver a “physiologic cocktail” of VEGF, TGF-β, interleukin-10, and extracellular vesicles that boosts angiogenesis, tempers excessive inflammation, and accelerates fibroblast and keratinocyte proliferation, thereby hastening re-epithelialization in chronic or irradiated wounds^[75,77,79]^. Early clinical experience is encouraging: same-day ADSC injections closed refractory chest-wall and sarcoma resection defects without local recurrence at 1-year follow-up, while autologous bone-marrow sprays reduced time-to-closure in irradiated scalp ulcers and head and neck free flap donor sites^[77,80]^.

These benefits, however, are counter-balanced by oncologic and regulatory concerns. Pro-regenerative cytokine milieus can inadvertently nurture dormant tumor clones; murine models show that mesenchymal stromal cells may home to residual neoplastic foci and stimulate growth through paracrine loops^[81]^. Heterogeneity and variable viability of bedside concentrates also raise infection, graft failure, and product-to-product inconsistency risks, while the potent immunosuppressive profile of some mesenchymal stem cells subsets could attenuate the desired pro-inflammatory anti-tumor environment created by ICIs^[82,83]^. Regulatory agencies therefore classify even “minimally manipulated” autologous tissue products as advanced-therapy medicinal products or human tissue-based products, mandating sterility testing, release criteria, and post-infusion surveillance, albeit under expedited pathways compared with culture-expanded or gene-edited cells^[80,84]^. Current expert consensus recommends deferring cell-based reconstruction until cancer remission has been firmly documented^[85]^, with multidisciplinary tumor-board approval for each case^[80,86]^. However, high-quality randomized trials with long oncologic follow-up remain essential to confirm safety while unlocking the regenerative potential of minimally manipulated cells for this uniquely vulnerable population.

**Palliative surgery under immunotherapy**: Advanced tumors can form complex fungating wounds associated with pain, infection, malodor, heavy exudate, and bleeding, which severely impair quality of life^[87]^. Palliative surgical interventions (such as tumor debulking or excision with skin grafting) are often undertaken to alleviate these distressing symptoms and improve patient comfort. Even without curative intent, debulking can reduce foul discharge and odor, decrease infection risk, relieve pain, and thereby potentially improve nutritional status and overall performance^[87]^. In patients receiving immunotherapy, careful timing of palliative surgery is crucial. Evidence suggests that surgery can be safely performed during ongoing immunotherapy in select cases^[88,89]^. For example, a retrospective series reported no major (grade 3–4) complications in 22 operations on patients treated with ICIs, indicating that immunotherapy may not need to be stopped perioperatively^[5]^. However, a head and neck cancer series found that patients receiving immunotherapy preoperatively had higher rates of wound dehiscence and donor-site complications^[1]^, whereas starting immunotherapy only after adequate wound healing did not impair recovery^[1]^. Thus, many clinicians advocate scheduling surgery between immunotherapy cycles (or temporarily pausing treatment) and resuming immunotherapy only after the surgical wound has adequately healed^[90,91]^. Expected risks of palliative surgery under immunotherapy include typical surgical morbidities (bleeding, infection, delayed healing) along with immune-related considerations. Checkpoint inhibitors themselves do not cause the myelosuppression seen with chemotherapy, but if irAEs necessitate high-dose corticosteroids, wound healing could be impaired^[72]^. Meticulous postoperative wound care is paramount. Management should include strict infection control and absorptive dressings for exudate, plus measures for odor control such as topical metronidazole or silver-based dressings^[92]^. Adequate analgesia and hemostatic techniques help manage pain and bleeding. Ultimately, judicious palliative surgery during immunotherapy can improve quality of life and help patients maintain dignity in advanced disease^[93]^.

**Future directions**: As immunotherapy remains an evolving field, ongoing and future clinical trials should incorporate detailed monitoring of wound healing and surgical outcomes to generate high-level evidence. Notably, in this review, although available evidence suggests acceptable wound outcomes with immunotherapy, most cited series are retrospective, single-center and include fewer than 100 patients, making publication bias and type-II error likely. Their favorable findings may therefore over-represent best case practice. Prospective, multiinstitutional registries and inclusion of unpublished negative results, especially from community hospitals, are needed to capture rare complications and provide generalizable risk estimates. Prospective data would help establish guidelines on perioperative immunotherapy management (e.g., an optimal interval between ICI and surgery, if any). Research into biomarkers might identify which patients are at higher risk for wound complications—for example, patients with certain cytokine profiles or underlying autoimmune markers could potentially be flagged. On the bioengineering front, intriguing possibilities include locally counteracting the immunotherapy’s effect in a wound (for instance, topical PD-L1 protein or gene therapy to a chronic wound in a patient on systemic anti-PD-1—a theoretical idea drawn from the pro-healing role of PD-L1)^[29]^. While such tactics are far off, they underscore the need to bridge oncology and wound healing research.

## Conclusions

Most cancer patients on immunotherapy heal surgical and accidental wounds without major issues. Large studies show no significant differences in surgical complication rates with ICIs. However, immune system alterations can sometimes delay healing or cause unique wound pathologies. Neoadjuvant immunotherapy before complex surgeries has shown higher wound complication rates in some cohorts, suggesting careful patient selection is needed. Immune-related adverse effects can directly cause wounds (like autoimmune blistering diseases), requiring immunosuppressive treatment and specialized care. Clinicians should watch impaired healing or unusual wound presentations. Early intervention—optimizing wound care, specialist consultation, or adjusting immunotherapy—prevents minor issues from becoming serious. A multidisciplinary approach involving oncologists, wound care specialists, and dermatologists is ideal. Patient education ensures prompt reporting of problems.

## Data Availability

Data sharing not applicable—no new data generated.
